# Fatty Acid-Binding Proteins Identification during the Evolution of Metabolic Syndrome: A Raman Spectroscopy-Based Approach

**DOI:** 10.3390/molecules28227472

**Published:** 2023-11-08

**Authors:** Guadalupe Donjuán-Loredo, Ricardo Espinosa-Tanguma, Edgar Guevara, María del Carmen Rodríguez-Aranda, Fabiola León-Bejarano, Karen Hernández-Vidales, Miguel Ramírez-Elías

**Affiliations:** 1 Facultad de Medicina, Universidad Autónoma de San Luis Potosí, Av. Venustiano Carranza 2405, Lomas los Filtros, San Luis Potosí 78210, Mexico; 2 Coordinación para la Innovación y Aplicación de la Ciencia y la Tecnología (CIACyT), Universidad Autónoma de San Luis Potosí, Av. Sierra Leona 550, San Luis Potosí 78210, Mexico; 3 Consejo Nacional de Humanidades, Ciencias y Tecnologías (CONAHCYT), Universidad Autónoma de San Luis Potosí, San Luis Potosí 78210, Mexico; 4 Facultad de Ciencias, Universidad Autónoma de San Luis Potosí, Av. Chapultepec 1570, Privadas del Pedregal, San Luis Potosí 78295, Mexico

**Keywords:** metabolic syndrome, fatty acid-binding proteins, biomarkers, Raman spectroscopy

## Abstract

Excess fat in abdominal deposits is a risk factor for multiple conditions, including metabolic syndrome (MetS); lipid metabolism plays an essential role in these pathologies; fatty acid-binding proteins (FABPs) are dedicated to the cytosolic transport of fat. FABP4, whose primary source is adipose tissue, is released into the circulation, acting as an adipokine, while FABP5 also accompanies the adverse effects of MetS. FABP4 and 5 are potential biomarkers of MetS, but their behavior during syndrome evolution has not been determined. Raman spectroscopy has been applied as an alternative method to disease biomarker detection. In this work, we detected spectral changes related to FABP4 and 5 in the serum at different points of time, using an animal model of a high-fat diet-induced MetS. FABP4 and 5 spectral changes show a contribution during the evolution of MetS, which indicates alteration to a molecular level that predisposes to established MetS. These findings place FABPs as potential biomarkers of MetS and Raman spectroscopy as an alternative method for MetS assessment.

## 1. Introduction

Obesity has associated diseases like insulin resistance, dyslipidemia, and hypertension, which contribute to the development of type 2 diabetes mellitus and cardiovascular diseases, and when combined (Table 1), they are known as metabolic syndrome (MetS) [1].

Lipids and numerous adipokines, cytokines, and hormones are essential mediators for signaling pathways related to MetS, and abnormalities in this mechanism occur at different levels and in various tissues [2]. In cells with active lipid metabolism, storage, mobilization, and transport alterations are produced, affecting signaling pathways [3,4]. The cells that present these alterations contain high levels of fatty acid-binding proteins.

Fatty acid-binding proteins (FABPs) belong to the group of cytosolic proteins that can reversibly bind with great affinity to lipophilic ligands, such as long-chain saturated, unsaturated fatty acids and other lipids. Also, FABPs help transport these lipids to sites or organelles inside the cell [5].

Its molecular weight is about 14–15 kDa, and it has a well-known three-dimensional structure: an antiparallel β-barrel conformation with ten strands arranged in two orthogonal β-sheets with five strands on each. The ligand-binding site is located in a cavity within the β-barrel, the entrance of which is oriented toward the N-terminal helix–loop–helix-shaped domain, where fatty acids bind. [5,6].

Nine isoforms of FABPs have been identified with a 15–70% sequence identity between them, and each isoform is expressed in specific tissues actively involved in lipid metabolism. It can be expressed in adipose tissue (A-FABP/FABP4/aP2), the brain (B-FABP/FABP7), the epidermis (E-FABP/FABP5/mal1), heart (H-FABP/FABP3), ileal (Il-FABP/FABP6), intestine (I-FABP/FABP2), myelin (M-FABP/FABP8), liver (L-FABP/FABP1), and testis (T-FABP/FABP9) [6,7].

Fatty acid-binding protein 4 (FABP4) is expressed mainly in white adipocytes, its primary source, and to a lesser extent in macrophages. It coordinates the inflammatory and metabolic pathways of these cells [8]. The adipocyte’s secretion of FABP4 is associated with lipolysis, stimulation of the sympathetic nervous system, and fasting at glucagon-like levels, acting as an adipokine [9]. Fatty acid-binding protein 5 (FABP5) is mainly expressed in the epidermis but is also found in adipocytes and macrophages of the hypodermis. The mechanism of FABP5 secretion remains unclear [10]. These cytosolic proteins have no typical secretory signal peptides in their sequence; thus, it releases into the blood in a nonclassical pathway [11,12].

MetS research, based on animal models and clinical studies with humans [13,14,15,16], has demonstrated that elevated serum levels of circulating FABP4 and FABP5 are associated with all the clinical parameters of MetS [17,18], also including fatty liver disease [8], atherosclerosis [19], renal dysfunction, cardiac dysfunction [20], and cardiovascular events [21], thus showing strong pathophysiological participation of these FABPs. Also, it has been demonstrated that genetic deficiency [22], antibody neutralization [23,24], or pharmacological inhibition of FABP4 and FABP5 [25] attenuates metabolic, cardiovascular, and inflammatory disorders. Due to their relevance, it has been proposed that FABP4 and FABP5 can be considered independent predictors of metabolic and cardiovascular diseases [8].

The detection of biomarker proteins in serum is performed using molecular biology methods, which require an antibody or fluorescent marker to identify and quantify them. New techniques, such as Raman spectroscopy, can be considered an alternative for analyzing FABPs. Raman spectroscopy has certain advantages that allow it to be regarded as an alternative method for biomedical applications [26,27]: its relatively fast technique; processing of biological samples is not necessary; it is non-destructive as long as the power, integration time, and wavelength of the laser are considered; it allows the detection of minimal changes at the molecular and structural level in tissues giving relevant information on pathologies [28]. In a previously published work [29], we acquired Raman spectra of FABP4 at different concentrations to evaluate Raman’s feasibility in identifying FABPs. In this work, we report FABP4 and FABP5 in a more detailed approach, identifying the molecular structure and spectral features to identify these proteins in serum. There are no studies related to the FABPs detection in serum with Raman spectroscopy; therefore, our project aimed to explore with this optical technique the detection of proteins in the serum of animals during the evolution of the metabolic syndrome (MetS). In the future, Raman spectroscopy could be used for FABPs detection in different tissues and fluids to diagnose MetS with a minimally invasive method.

## 2. Results

### 2.1. Animal Model of MetS

Animals with a high-fat diet (HFD) intake showed a higher percentage of fat and total body fat with respect to total body weight from 8 weeks, this difference is significant at 18 weeks and the rest of the weeks compared to the control diet group. The abdominal circumference of MetS animals is higher than that of the Controls, although it does not have statistical significance. The Lee index measures the degree of obesity in rodents and is calculated using the cubic root of the total body weight (g) divided by the snout-anus length (cm). It is considered normal weight when the value is <0.300, and rodents are classified as obese when there is a value ≥0.300 [30]. Our MetS model has obesity from 18 weeks based on this index.

According to the American Heart Association 2017 criteria [31], the 8-week MetS animals have high blood pressure, the 18-week ones have hypertension stage I, the 28-week ones have hypertension stage II, and the 52-week ones have hypertension stage I compared with the control group. In general, fasting glucose levels in the obese group remain significantly higher than in the control group but do not meet the criteria for diabetes mellitus diagnosis [32]. Serum total cholesterol showed high statistically significant levels in the MetS group in all weeks except 28 weeks. Serum triglyceride levels showed a significant reduction in the 8-week MetS group, eventually showing a gradual rise in the same group and being highly significant at the end of 52 weeks. HDL cholesterol has a well-recognized cardioprotective function, whose serum levels are considered acceptable when they are >35 mg/dL and optimal >40 mg/dL [33]. Both groups maintained similar levels throughout the study, except at 28 and 52 weeks, when there was a reduction in the MetS group compared to the control group.

The serum concentrations of FABP4 in the MetS groups of 8 and 52 weeks showed significantly high levels. They maintained this elevation at 18 and 28 weeks compared to the control group but without statistical significance. In FABP5, we observed a significant increase at 8 weeks with a decrease at 18 weeks, but from 28 weeks, its levels increased significantly. They were maintained until 52 weeks in the MetS group when the pathology was established (Table 2).

The fasting glucose results obtained in the different weeks of the HFD group were inconclusive for the clinical diagnosis of diabetes mellitus type 2 or alterations in glucose metabolism. For this reason, we decided to perform other appropriate methods for assessing glucose metabolism, such as OGTT and ITT [32].

**Figure 1:**
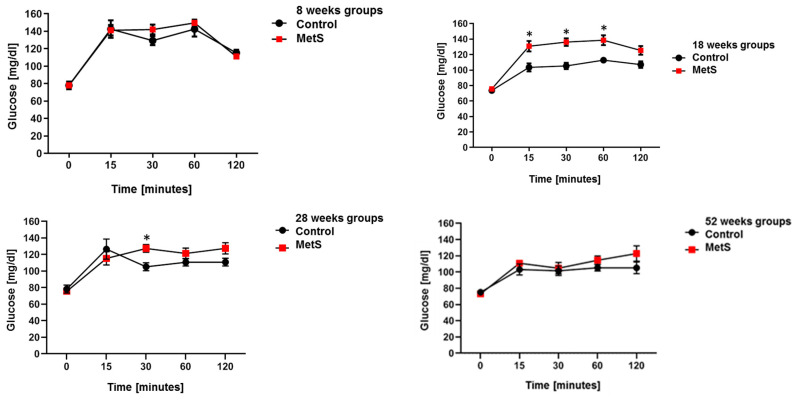
Results of oral glucose tolerance curves (2 g/kg, 50% glucose) for each time point (*n* = 8, * *p* < 0.05).

The OGTT at 8 weeks does not show any difference between groups. At 18 weeks, the difference is significant since the experimental group shows an increase in serum glucose levels at different times. This difference was not observed in the subsequent 28 and 52 weeks, probably due to an improvement in insulin sensitivity as compensation for the initial metabolic imbalance with the diet (Figure 1).

**Figure 2:**
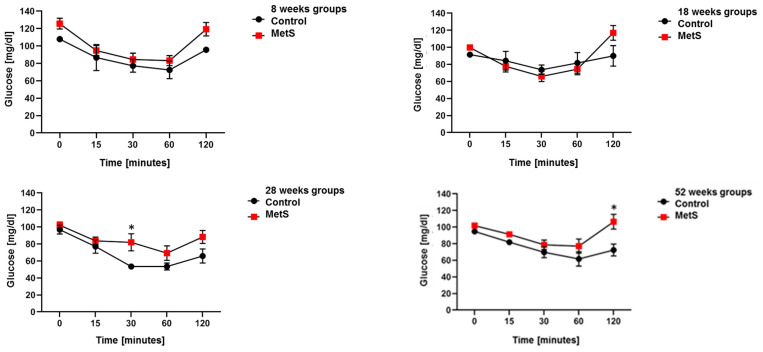
Results of response curve to insulin (0.75 U/kg) for each time point (*n* = 8, * *p* < 0.005).

The ITT results of the MetS groups show elevated blood glucose levels compared to the control group, being evident at the last point of 120 min. Thus, it indicates insulin resistance in insulin-sensitive tissues and makes it difficult to maintain levels in the physiological range (Figure 2).

### 2.2. Raman Spectrum FABP4 and FABP5 In Vitro Analysis

**Figure 3:**
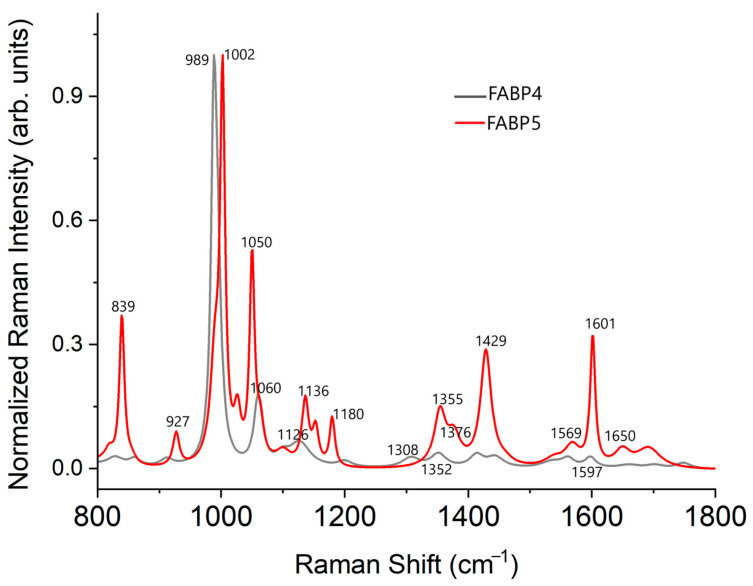
Raman spectra from the proteins FABP4 and FABP5. Each spectrum was normalized and baseline corrected. To obtain the peaks reported in Table 3, we deconvolute the Raman spectrum of FABP4. Figure 4 shows the peak deconvolution for the FABP4 protein.

Figure 3 shows each Raman spectra from FABP4 and FABP5 in the measured region: 400 to 1800 cm^−1^; this region is known as the fingerprint area [34]. The spectrum from FABP5 has a better peak resolution, while the FABP4 presents more overlapped regions and less intensity; however, both spectra have similar shapes.

**Figure 4:**
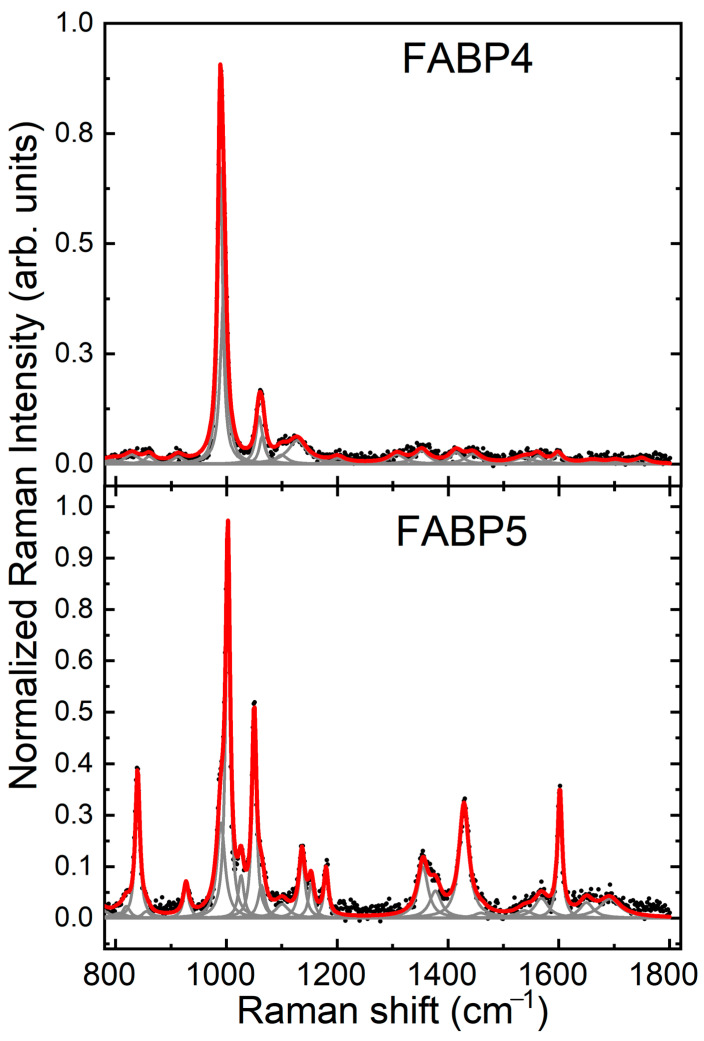
Deconvolution of the Raman peaks for FABP4 and FABP5 proteins.

Table 3 (below) shows the Raman bands observed in each spectrum and their corresponding vibrational modes. The strongest vibrations and the sharper ones are resalted. Most fingerprint bands come from CH, CH_2,_ and CH_3_ vibrations [35].

**Table 3:** Vibrational modes of FABP4 and FABP5.

FABP4		FWHM	FABP5		FWHM	Vibration Mode	Amino Acid
			449	vw	37.7	Cys; δ(backbone)	Cys and Met
457	m	26.7				δ(C-C-C-C); ν(S-S)	Phe, Cys, and Met
533	vw	39.4	528	vw	21	δ(C-C=C); δ(ring); ν(S-S)	Phe, Cys, and Met
			612	w	9.7	δ(O-C=O)	Phe
627	w	26.1	631	sh	26.2	Symmetric ring breathing	Phe
678	vw	29.2	674	w	8.3	ν(C-S)	Cys and Met
707	vw	34.2	713	vw	18.5	ν(C-S)	Cys and Met
			766	vw	33.2	ν(C-C); ζ(CH_3_)	Met
			819	sh	16.2	ν(C-C)	Tyr and His
828	vw	33.6				ω(CO_2_); τ(CO_2_)	Aliphatic amino acids, Tyr
			839	m	9.6	ring breathing	Tyr
860	vw	22.3	855	vw	17.6	ω(C-H); τ(C-H)	His, Arg
912	vw	26.5				ν(C-C-N)	Asp and Glu
			927	vw	11.8	δ(C-C); ν(C-C)	Phe
988	vs	10.3	990	sh	17.6	C-C	Arg and Lys
			1002	vs	9.6	δ(ring)	Phe
			1026	w	12.6	δ(ring)	Phe
			1050	s	9.6		His
1060		14.6					His
1064	sh	13	1063	sh	14.4	C-C, ν(C-N), δ(ring), NH_3_	Arg and Lys
1099	vw	25.8	1100	vw	30.0	ζ(CH_2_), ν(C-N), ζ(CH_3_)a, N-C-H,	His, Pro, Met, and Cys
1127	w	41.8				ω(NH_3_^+^)	Asp and Glu
			1136	w	12.8	τ(CH_2_); ν(CN); ν(CCH); δ(COH); ν(CC)a; ω(NH_3_); τ(CH_3_); ν(C-C)	Gly, Phe, Tyr, Trp, His, Gly, Lys, Leu, and Iso
			1153	w	11.9	δ(C-H)	Tyr, Trp, and Phe rings
			1180	w	9.6	δ(COH), ν(CC), δ(CH_3_), τ(NH_3_)	Tyr and Leu
1200	vw	29.5				symmetric stretching	Aromatic amino acids
1307	vw	34.8				ν(C-N); ν(N-H); δ(backbone)	Amide III
1352	w	30.9	1355	w	22.5	ν(C-C); δ(CCH); ω(CH_2_); δ(CH); δ(rings)	Arg, Lys, Leu, and Trp
			1377	vw	25.7		Trp
1414	vw	27.5				ν(CH3); δ(CH_3_); δ(CH_2_)	Lys, Aliphatic amino acids
			1429	m	20.9	ν(NH_2_); ν(COO-); δ(CH_2_)	Asp, Glu, and Trp
1444	vw	33.9				δ(CH_3_)a; δ(CH_2_); δ(CH_3_); δ(COO-); ν(C-C)	Met, Leu, Trp, Gly, Leu, and His
			1459	vw	31.2	β(CH_3_)a	Leu
1536	vw	42.6	1538	vw	36.6	ν(C-N); β(N-H)	
1562	vw	24.4	1568	vw	30.5		Trp
1598	vw	21.8	1601	m	9.6	ν(COO-)a; ω(OH_2_); ν(C-O)	Phe and Ala
			1650	vw	30.3	ν(C=O); δ(NH_3_)	Amide I, α-Helix, and β-sheet
1660	vw	42.3					
1702	vw	34.6	1692	vw	46.6	ν(C=O); σ(NH_3_)	
1749	vw	29.6					

ν: stretching; δ: deformation; β: bending; ζ: rocking; ω: wagging; τ: twisting; σ: scissoring; a: asymmetric.

In both spectra, the most intense vibration is located around 1000 cm^−1^, which corresponds to the ring deformation of phenylalanine (Phe); however, in the protein FABP4, the precise position of the peak is 988 cm^−1^; this blue shift could indicate vibrations that correspond to the amino acids lysine (Lys) and arginine (Arg) [36]. It is noticeable that the most intense peak in FABP5 has a shoulder at 990 cm^−1^; therefore, we can conclude that these strong peaks in both proteins are a combination of two vibration modes: the ring deformation of Phe and the vibrations of Lys and Arg. 

The FABP4 has the second strongest peak at 1060 cm^−1^, which could be assigned to histidine (His). For the FABP5, a shoulder is observed at 1064 cm^−1^, related to vibrational modes of amino acids Arg and Lys. Also, it is observed a stronger Raman band at 1050 cm^−1^, which corresponds to His [36,37]. 

Cystine (Cys) and methionine (Met) are sulfur-containing amino acids that cause vibrations at 457 and 528 cm^−1^ due to S-S bonds. The small peak at 766 cm^−1^, only visible for FABP5, is assigned to C-C stretching and CH_3_ rock of Met. Other Raman bands from sulfuric bonds are 449, 533, 674, 713, 1100, and 1444 cm^−1^ [34,36]. 

The aromatic amino acids Phe, Trp (tryptophane), and Tyr (tyrosine) cause the following Raman bands: 1136, 1153, and 1200 cm^−1^. The vibration at 839 cm^−1^ is considered a marker of Tyr; the peaks 1377 and 1568 cm^−1^ have been assigned to Trp; and Phe contributes to the vibration bands around 612, 630, 927, 1002, 1026, and 1601 cm^−1^ [34,36,38]. 

The Amide III and Amide I regions are clear in the FABP5 spectrum and barely noticeable in the FABP4 Raman spectrum. These regions allow us to confirm the secondary structure of the proteins since they represent the vibrations of α-helixes and β-sheets conformations [38].

### 2.3. Identification of FABP4 Spectral Features

**Figure 5:**
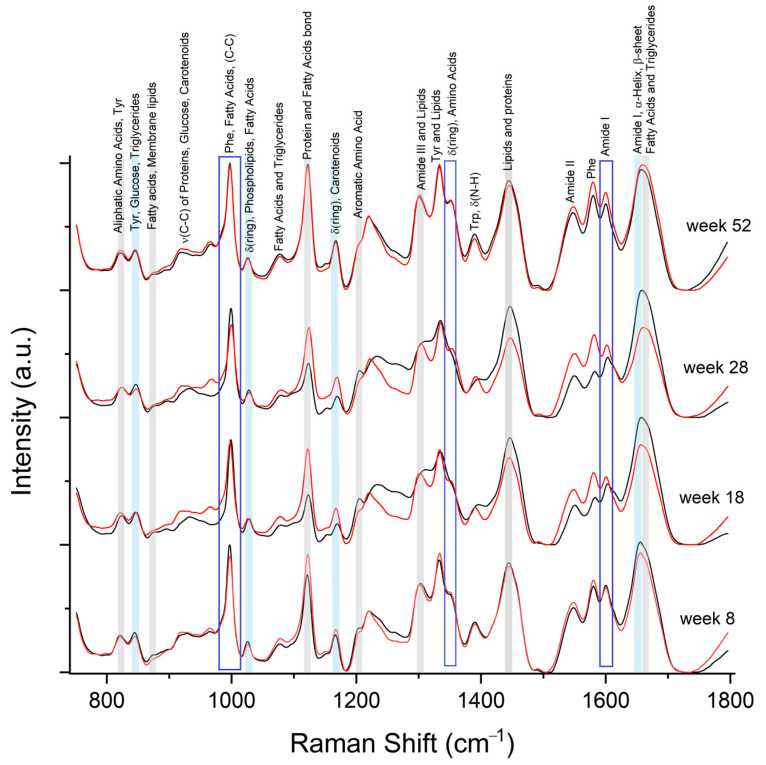
Mean Raman spectrum from control serum (black line) and MetS (red line) at weeks 8, 18, 28, and 52. The grey marker highlights the bands that coincide with bands in the Raman spectrum from FABP4, the blue marker highlights the coincidences in FABP5, and the blue rectangles correspond to bands where both FABP have Raman bands. All the spectra were normalized to the unit area to allow direct comparison.

Using the FABPs Raman spectrum as a reference, we identify its spectral features in serum samples. Figure 5 shows the mean Raman spectra of Control and MetS at different temporal points.

The Raman bands at 828 cm^−1^ and 860 cm^−1^ from FABP4 could be components of the vibrations well defined at the serum spectra at 823 cm^−1^ and 875 cm^−1^, respectively; these bands correspond to aliphatic amino acids and tyrosine (Tyr). Also, the following have been reported: 877 cm^−1^ as C-O-O vibration for fatty acids and ν_as_(N^+^)(CH_3_)_3_ in membrane lipids [39].

The Raman spectrum from FABP4 has the most intense band at 989 cm^−1^ related to carbon bond vibrations (C-C), while the Raman spectra from serum observed a strong band at 999 cm^−1^. However, this vibration could signal the presence of the FABP since it is also a vibrational marker for fatty acids [39]. It suggests that the strong peak at 999 cm^−1^ in the serum spectra is the vibrational marker for the FABPs and their union with fatty acids. 

In the serum, a high-intensity Raman peak is located at 1122 cm^−1^ assigned to Glucose, fatty acids, triglycerides, and membrane lipids; but more importantly, this band could be a sign of the union in the serum since the FABP4 has a Raman band at 1127 cm^−1^. In almost all Raman spectra from serum, a prominent shoulder at 1202 cm^−1^ can be associated with symmetric vibration of aromatic amino acids in the FABP4. Another vibration in both Raman spectra is 1303 cm^−1^, part of Amide III, and reported for fatty acids, triglycerides, cholesterol, and membrane lipids [39]. 

Around 1350 cm^−1^, another intense band is present for the serum spectra. It coincides with a weak vibration in the Raman spectrum of FABP4, associated with C-H, C-C, and ring vibrations from several amino acids. At 1445 cm^−1^, a broad, intense band in the serum spectra is observed, as in FABP4. This region is a marker for membrane lipids, fatty acids, triglycerides, and cholesterol assigned to the vibrations σ(CH_2_/CH_3_), δ(CH_3_), δ(CH_2_), δ(COO-), and ν(C-C). At 1600 cm^−1^, another band is present in serum, also found in FABP4, and usually associated with the region Amide I for proteins. Finally, the Raman band at 1656 cm^−1^, weakly present in FABP4 but intense in serum spectra, is assigned to the Amide I region and secondary structure of the proteins: α-helixes and β-sheets; both structures are FABP4 characteristics. Additionally, this band has been reported as a marker of ν(C-C) for fatty acids, triglycerides, cholesterol, and membrane lipids [40]. 

### 2.4. Identification of FABP5 Spectral Features

The FABP5 has a Raman peak of 840 cm^−1^, which can be associated with Tyrosine. In serum, this peak are found at 845 cm^−1^, Also, lipids are a vibration for COO in triglycerides and close to characteristic vibrations C-H bond of Glucose. 

FABP5 has an intense peak at 1002 cm^−1^ and a shoulder at 990 cm^−1^, which we can observe at 999 cm^−1^ in serum. These vibrations can be overlapped with the vibrations from the FABP4 and are associated with the deformation of aromatic rings in Phenylalanine (Phe), C-C bonds, and fatty acids. At 1028 cm^−1^, all Raman spectra from serum show a clear weak peak. This peak is also present in FABP5 assigned to ring deformation; other associations have been reported for phospholipids and β(CH) for fatty acids. A Raman band is distinguishable at 1154 cm^−1^, also present in FABP5, which is associated with the deformation of aromatic amino acids and carotenoids. 

FABP5 spectrum also presents a Raman band at 1600 cm^−1^, associated with the region Amide I for proteins, which resembles the secondary structure of the proteins. The band at 1650 cm^−1^, well visible in both spectra, is also part of the Amide I region; it also has close vibrations of lipids: fatty acids at 1653 cm^−1^, triglycerides at 1656 cm^−1^, and 1654 cm^−1^ for membrane lipids. 

### 2.5. Spectral Differences Related to FABP4 and 5 in Serum

**Figure 6:**
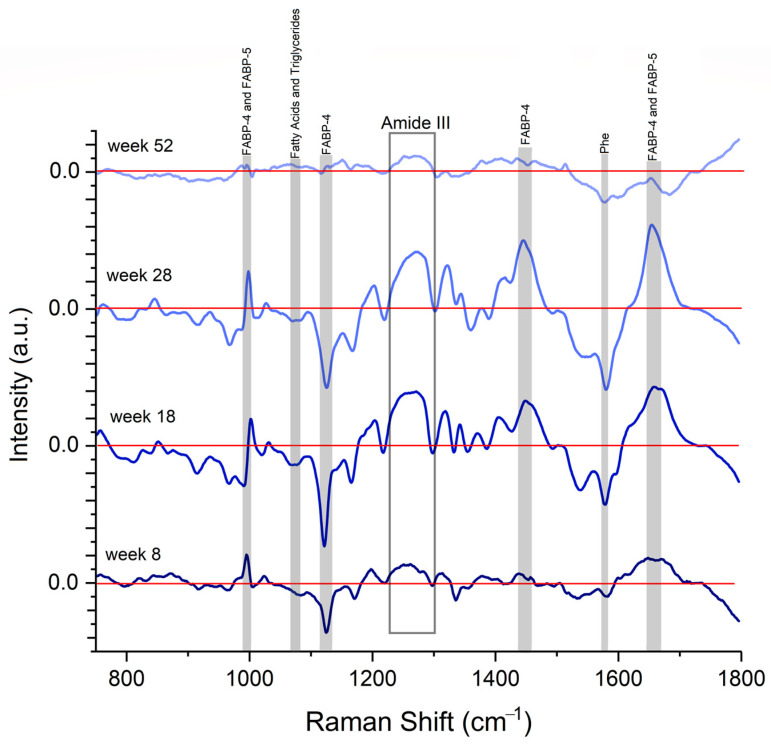
Difference spectra (Control minus MetS) for 8, 18, 28, and 52 weeks. The most relevant differences related to FABP4, FABP5, and lipids are highlighted in grey. Raman spectra were subtracted in the ratios 1:1. All the spectra were normalized to the unit area before subtraction.

We calculate the differential spectra between the Control and MetS groups. Figure 6 shows the difference spectrum (Control minus MetS) calculated for 8, 18, 28, and 52 weeks. Positive values imply a stronger intensity in the control spectrum, and negative values indicate more intense vibrations in the MetS spectrum. Values close to zero indicate similar Raman peak intensity on both spectra.

The Raman bands at 990, 1445, and 1650 cm^−1^ have a similar behavior: they all have positive values and increase when they evolve to 18 and 28 weeks. Then, their values decrease to close to zero at 52 weeks. This means that amide III, FABP4, and FABP5 are higher in controls at 8, 18, and 28 weeks. FABP-4 and FABP-5 increase together at 18 and 28 weeks and have similar values in both groups at 52 weeks. Amide III maintains higher controls for all weeks, increasing at 18 and 28 weeks. It is noted that at 18 and 28 weeks, the 800–1000 cm^−1^ spectral region from MetS shows alterations related to proteins and lipids. 

On the other hand, the bands at 1080, 1122, and 1580 cm^−1^ have negative values, which means these bands are strong in MetS. They show an increased negative value at 18 and 28 weeks and a value close to zero at 52 weeks. It means fatty acids, phenylalanine, and FABP4 are higher in MetS at 8, 18, and 28 weeks. Fatty acids and FABP4 increase at 18 and 28 weeks and have similar values in both groups at 52 weeks. Phenylalanine increases at 18 and 28 weeks and maintains a higher intensity in MetS.

### 2.6. PCA-SVM

**Figure 7:**
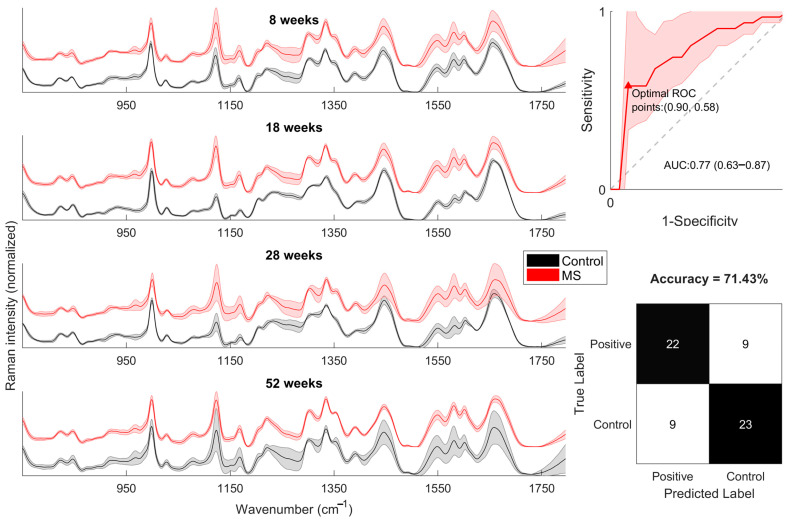
Mean serum Raman spectra from Controls (black) and MetS (red) for 8, 18, 28, and 52 weeks (**left**). ROC and confusion matrix of PCA-SVM model (**right**).

Additionally, we apply chemometrics to our dataset. First, we use SVM to classify Raman spectra of control and SM groups from 8, 18, 28, and 52 weeks. The confusion matrix shows a similar number of samples correctly classified for both groups, with an accuracy of 71.43%. The ROC curve analysis shows an AUC value of 0.77 (0.63–0.87) and an optimal point of Sp = 0.90 and Se = 0.58, as depicted in Figure 7. 

**Figure 8:**
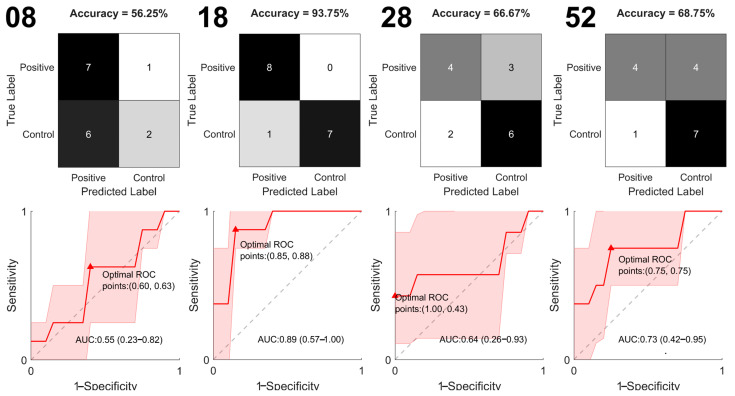
PCA-SVM confusion matrices (**top**) and ROC curves (**bottom**) for serum Raman spectra of controls and Mets for 8, 18, 28, and 52 weeks.

Later, we apply PCA-SVM to all spectra and analyze each temporal point (8, 18, 28, and 52 weeks). The result of SVM is shown in Figure 8. The confusion matrix shows that All SM samples were correctly classified for 18-week spectra, with an accuracy of 93.75%. The lowest accuracy was obtained in the 8-week spectra, which is expected because SM is not entirely developed; therefore, no significant differences with controls can be found. According to ROC curves, the 18-week spectra show the highest AUC value of 0.89 (0.57–1.0) and an optimal point of Sp = 0.85 and Se = 0.88. 

## 3. Discussion

We used HFD in an animal model to mimic all aspects of the human MetS and its complications, such as central obesity, alterations in glucose metabolism, insulin resistance, elevated blood pressure, and dyslipidemia [41,42]. Those are accepted risk factors that increase the incidence of cardiovascular diseases and type 2 diabetes [8].

Different fat-rich diets have been used in the range of 20% to 60% fat [30]. In our work, we employ approximately 40% animal fat added, which leads to a gradual evolution of the MetS, being established at 52 weeks.

A high concentration of FABP4 and 5 was an independent predictor for the development of MetS, according to follow-up studies in the Asian population [13,14,16]; however, the relationship between elevated levels of these proteins and their relationship with the gradual appearance of clinical parameters has not been established throughout the evolution of the MetS. 

The increased levels of FABP4 in circulation may be due to one or all of the following three factors: (1) the increased production of adipocytes as the primary source, given the positive correlation with a high amount of adipose tissue [4]; (2) the increased secretion mediated by increased lipolysis secondary to altered glucose metabolism in liver or activation of the sympathetic nervous system dependent on beta-adrenergic receptors [17]; (3) a low clearance from the circulation, probably due to renal damage caused by obesity [43,44].

Our results show an increase in serum concentrations of FABP4 at 8 weeks in the MetS group due to a higher amount of adipose tissue, which is the first verifiable sign in this group of animals. The increase is present at 18 and 28 weeks but is not significant, accompanied by altered blood pressure and a continuous increase in body fat. At 52 weeks, when the MetS is present in a chronic way, the levels are significantly high, corresponding even with dyslipidemia. This protein level elevation from the beginning of the diet is observed even before obvious signs of cardiovascular and metabolic alteration are detected. On the other hand, FABP5 serum concentrations rise gradually throughout the protocol, suggesting a role in the development of MetS, but this serum behavior is more constant in its elevation. In the control groups, the levels of both proteins do not change significantly, even because of the age of the animals (Table 2).

Our data suggest that the serum behavior of FABPs allows them to be positioned as possible biomarkers of MetS and associated pathologies even before it is established.

Once the MetS were established with routine tests, we used Raman spectroscopy to detect the FABPs in serum and see if they corresponded with the parameters previously obtained in the animal model.

According to the Raman analysis, it was possible to differentiate between FABP4 and FABP5, despite their spectral similarity (Figure 4). This similarity is expected because both proteins are isoforms of the FABPs [5,7]. Their Raman spectra show vibrational model characteristics for each FABP, with the main differences in the Amide I and Amide III regions, which are related to the secondary structure of proteins (Table 3). From the serum Raman spectra, we can observe that bands related to FABP4 and FAB5 change in intensity at 18 and 28 weeks, while at 8 and 52 weeks, these FABP-related bands have similar intensity Figure 6). This behavior is observed clearly when the difference spectrum is calculated, suggesting that FABP4 and 5 contribution is high when the MetS induced by diet is in course with a developing metabolic and cardiovascular disorder. 

During the evolution and establishment of MetS (18 to 28 weeks), FABPs bind free fatty acids in the cytosol to mobilize them to undergo conformational changes. Instability in its structure has been previously observed in these proteins [45], perhaps to increase the effectiveness in transporting and storing lipids in tissues. At 52 weeks, no spectral differences are seen, suggesting that the animal’s organism adapts to these pathological conditions, which we surely would not see in acute disease. It could be further confirmed with techniques such as crystallography.

According to PCA-SVM, the classification performance is higher at 18 and 28 weeks and lower at 8 and 52 weeks, which agrees with the band analysis approach results. It suggests that the higher performance of the classifier at 18 and 28 weeks is due to spectral differences caused by changes in FABPs. On the other hand, the lower perforce is due to the contribution of FABPS in both groups, reducing the spectral difference between groups and thus reducing the classifier’s performance.

## 4. Material and Methods

### 4.1. Animals and Experimental Design

All animal experiments were performed in accordance with relevant of the approved guidelines and regulations of the Internal Committee for the Care and Use of Laboratory Animals of the School of Medicine of Autonomous University of San Luis Potosí (protocol No. BGFMUASLP-07-19). The reporting in the manuscript follows the recommendations in the ARRIVE guidelines.

Male wild-type Wistar rats (110 ± 10 gr) were kindly provided by the bioterium facility of the School of Medicine of the Autonomous University of San Luis Potosí. All rats were individually housed in acrylic cages, with constant conditions at 22 °C with a 12:12 h light/dark cycle, and fed food and water ad libitum.

A total of 64 Wistar rats were randomly divided into eight groups for different time points: Control groups of 8, 18, 28, and 52 weeks (Ctrl, *n* = 8) and metabolic syndrome groups of 8, 18, 28, and 52 weeks (MetS, *n* = 8).

Control groups were fed a standard diet (LabDiet 5001), while MetS groups were fed a high-fat diet (HFD) manufactured in our laboratory. The nutrient content of HFD was measured by bromatological analysis; the diet contained proteins (11.46%), carbohydrates (36.3%), lipids (40.72%), humidity (6.76%), and fiber (4.76%).

After each time point (8, 18, 28, and 52 weeks), all rats were sacrificed at 8 am under general anesthesia (intraperitoneal injections of 50 mg/kg sodium pentobarbital).

### 4.2. Blood Pressure Measurement

Using a CODA tail-cuff system, a non-invasive method was used to acquire systolic, diastolic, and mean blood pressure (Kent Scientific Corporation, Torrington, CT, USA). All measurements followed the manufacturer’s recommendations to avoid stressful situations in the animals, a noise-free place where rats were previously familiarized with the handling and instrumentation. Blood pressure was taken simultaneously in the morning, with ten repetitions for each animal, and the average was calculated.

### 4.3. Oral Glucose Tolerance Test (OGTT) and Insulin Tolerance Test (ITT)

On the test day, all rats were weighed, and a blood glucose sample was taken via punction on the tail at 0 min and measured with a glucometer.

A week before the OGTT, ITT was performed in non-fasted rats injected intraperitoneally with insulin (0.75 U/kg body weight). The food was immediately removed, but the water was left ad libitum, and blood glucose levels were tested at the tail at 15, 30, 60, and 120 min after injection.

On the OGTT day, the animals fasted overnight only with water ad libitum, and an orogastric catheter was used to administer a 2 g/kg body weight of 50% glucose. Finally, the blood glucose levels were tested at the tail at 15, 30, 60, and 120 min.

### 4.4. Serum and Tissue Collection

All rats were sacrificed at the end of 8, 18, 28, and 52 weeks, and blood samples were collected via cardiac puncture and placed in sterile tubes. Serum samples were separated by centrifugation at 2500 rpm for 15 min and stored in three different vials. One vial was sent to a clinical laboratory to quantify total cholesterol, triglycerides, and HDL-c; the remaining two were stored at −70 °C for ELISA and Raman spectroscopy measurements of FABP4 and FABP5.

Adipose tissue was extracted from different anatomical deposits, epididymal, retroperitoneal, visceral, and pericardial; the total amount of adipose tissue was obtained to obtain the fat percentage in relation to weight.

### 4.5. Serum ELISA Assay for FABP4 and FABP5

The serum collected and stored at −70 °C is placed at room temperature until thawed, vortexed, and slightly centrifuged. Concentration gradients are prepared from the working solution of the ELISA kit for the standard curve of each protein FABP4 and 5. The protocol is carried out according to the supplier’s specifications (Novus Biologicals Rat FABP4/A-FABP ELISA Kit NBP2-82484, MyBioSource Rat FABP5/E-FABP ELISA Kit MBS2882161).

### 4.6. Raman Spectra of FABP4 and FABP5

Lyophilized recombinant proteins were purchased (10 μL FABP4 of Novoprotein, 2 μL FABP5 of MyBiosource). FABP4 and FABP5 were centrifuged and reconstituted with 100 μL and 200 μL of milliQ water, respectively. Then, a concentration of 100 ng/mL was calculated, and 5 μL was taken from every FABP solution, drop-coated on an aluminum substrate, and allowed to dry at room temperature for 15 min. The Raman spectrum was acquired using the drop-coating technique recommended for protein solutions at very low concentrations.

### 4.7. Raman Spectra Serum

Raman spectra of 8, 18, 28, and 52 weeks of serum samples were thawed, vortexed, and briefly centrifuged. An amount of 5 μL of each previously labeled sample was taken and drop-coated on an aluminum substrate. Raman measurement was performed in a semi-wet drop.

All Raman spectra were recorded using an XPLORA ONE Raman spectrometer system (Horiba Scientific, Piscataway, NJ, USA) and 1800 lines per mm grating and spectral resolution of 0.8 cm^−1^ per pixel. A 20× objective lens (N.A = 0.4) was used to focus the laser beam on the ring of the drop coating deposited samples. A 532 nm laser wavelength was used, and the power at the sample’s surface was 35 mW. Triplicate measurements with 5 s of integration time were acquired in the “coffee ring” formed for each drop coated.

### 4.8. Statistical Analysis

All values of the MetS animal model are expressed as mean ± standard error of the mean (SEM). The Shapiro–Wilk normality test was applied. Differences between the 2 groups (control vs. MetS) for each temporal point were assessed using a *t*-test. The differences were considered statistically significant when * *p* < 0.05. All statistical analyses were performed using GraphPad Prism 8.0 software.

### 4.9. Data Preprocessing

Before data analysis, Raman spectra were preprocessed to remove noise and autofluorescence using the modified Vancouver Raman algorithm (mVRA). The mVRA is a method based on the Vancouver Raman algorithm (VRA) and empirical mode decomposition (EMD) for denoising Raman spectra of biological samples, composed of two main steps: signal filtering and polynomial fitting. This algorithm has the advantage of using EMD as an adaptive parameter-free signal processing method and an automated polynomial degree selection [46]. All the spectra were normalized to the unit area. Preprocessing was performed using in-house scripts written in R. Then, the deconvolution of FABP Raman peaks was performed using the Fityk 1.3.0 software to perform the Raman peak assignments. The fitting process was performed using the Fityk program (V 1.3.0). All spectra were fitted using Lorentzian shape functions.

### 4.10. PCA-SVM Model

Raman spectra classification was performed using a combined principal component analysis and support vector machine (PCA-SVM) model. PCA is a multivariate analysis technique used for the dimensional reduction of data. PCA constructs new variables that explain most of the variance in the data. We can use the variables that contain most of the variance (information) to identify differences between samples or relationships between variables, as well as using them as an input to classification algorithms. SVM is a classification algorithm that finds an optimal linear hyperplane that separates the classes. The SVM was used with a radial basis function kernel, and its optimal hyperparameters were found through a Bayesian optimization process. In our PCA-SVM model, PCA scores were used as inputs for the SVM. The model performance was assessed using overall accuracy (ACC), sensitivity (Se), specificity (Sp), and area under the receiver-operating characteristic (ROC) curve (AUC).

## 5. Conclusions

MetS includes a recognized variety of metabolic and cardiovascular alterations, increasing mortality risk. Detecting it in time allows appropriate therapeutic interventions, and having an established biomarker is a desirable objective. Early detection with alternative non-destructive methods, such as Raman spectroscopy, can be beneficial. In our work, we observed the feasibility of both FABPs as potential early markers of MetS and Raman Spectroscopy as a powerful tool for MetS studies.

## Figures and Tables

**Table 1:** Three of the following five clinical parameters are required to diagnose MetS.

Clinical Parameters	Measure
Central obesity; abdominal circumference	Men ≥ 90 cm, women ≥ 80 cm
Triglycerides	≥150 mg/dL
HDL cholesterol	Men ≤ 40 mg/dL, women ≤ 50 mg/dL
Hypertension	≥130/85 mmHg
Fast hyperglycemic	≥110 mg/dL

**Table 2:** Results of anthropometric and biochemical parameters quantified to determine the presence of MetS (*n* = 8, * *p* < 0.05).

	8 Weeks		18 Weeks		28 Weeks		52 Weeks	
Parameter	Control	MetS	Control	MetS	Control	MetS	Control	MetS
% Fat	3.9 ± 0.2	5.2 ± 0.3	3.8 ± 0.4	7.9 ± 0.8 *	5.3 ± 0.9	9.1 ± 1.1 *	6 ± 0.4	11.2 ± 1.4 *
Total body fat (g)	14.5 ± 1.3	14.6 ± 2	17.2 ± 2.2	36.4 ± 5.7 *	27.8 ± 5.6	49.3 ± 8 *	35.5 ± 3.8	90.1 ± 15.6 *
Lee index	0.290 ± 0.002	0.284 ± 0.003	0.296 ± 0.004	0.300 ± 0.004	0.317 ± 0.004	0.311 ± 0.004	0.305 ± 0.003	0.323 ± 0.008
Abdominal circumference (cm)	18.3 ± 0.6	15.9 ± 0.6	19.2 ± 0.6	20.9 ± 0.9	21.2 ± 0.5	23 ± 0.8	23.2 ± 0.7	26 ± 1.5
Systolic blood pressure (mmHg)	97 ± 6.8	122.9 ± 5.1 *	87.9 ± 18.2	132.9 ± 30 *	107 ± 5.4	142.8 ± 4.7 *	102 ± 4.7	132 ± 9.3 *
Diastolic blood pressure (mmHg)	65.1 ± 4.7	84.2 ± 3.8 *	61.9 ± 13.2	84.8 ± 19.9 *	64.6 ± 5	90.4 ± 4.6 *	72.8 ± 3.8	86 ± 7.4
Mean blood pressure (mmHg)	75.4 ± 5.4	96.8 ± 4.2 *	70.3 ± 9.8	100.6 ± 23.1 *	78.6 ± 4.6	108.3 ± 4.6 *	82.3 ± 3.7	101 ± 7.9 *
Fast Glucose (mg/dL)	108 ± 2.7	125.7 ± 6.2 *	91.5 ± 2.1	99.7 ± 2.1	92.2 ± 4.4	108 ± 3.2 *	96.3 ± 2.2	104.2 ± 2 *
Total Cholesterol (mg/dL)	53.1 ± 5.4	69.5 ± 8.6 *	49.1 ± 3.8	59.6 ± 6.4 *	53.8 ± 4.5	56.7 ± 5.2	76.6 ± 10.1	105.3 ± 13.6 *
Tryglicerides (mg/dL)	79.1 ± 6.9	57.4 ± 5.7 *	60.4 ± 12.9	82.9 ± 8.4 *	48.8 ± 6.2	73.2 ± 11.2 *	134.1 ± 21	273.4 ± 63.7 *
HDL Cholesterol (mg/dL)	23.1 ± 2	25.5 ± 2.3	17.9 ± 2.6	19.4 ± 2.7	22.2 ± 3.1	15.5 ± 3.5	32.4 ± 5.4	21 ± 4.6
FABP 4 ng/mL	24.6 ± 2	36 ± 2.2 *	26.3 ± 2.3	31.8 ± 1.1	22.6 ± 2.3	29 ± 0.8	29.6 ± 2.5	37.4 ± 2 *
FABP 5 ng/mL	1.9 ± 0.5	3.4 ± 0.9	1.9 ± 0.8	2.8 ± 1.6	2.3 ± 0.5	6.1 ± 0.8 *	1.9 ± 0.8	5.5 ± 0.9 *

## Data Availability

The datasets used and/or analyzed during the current study are available from the corresponding author upon reasonable request.
